# Serological response to SARS-CoV-2 vaccination in multiple sclerosis patients treated with fingolimod or ocrelizumab: an initial real-life experience

**DOI:** 10.1007/s00415-021-10663-x

**Published:** 2021-06-26

**Authors:** Guerrieri S., Lazzarin S., Zanetta C., Nozzolillo A., Filippi M., Moiola L.

**Affiliations:** 1 Neurology Unit, IRCCS San Raffaele Scientific Institute, Milan, Italy; 2 Neurorehabilitation Unit, IRCCS San Raffaele Scientific Institute, Milan, Italy; 3 Neurophysiology Unit, IRCCS San Raffaele Scientific Institute, Milan, Italy; 4 Neuroimaging Research Unit, Division of Neuroscience, IRCCS San Raffaele Scientific Institute, Milan, Italy; 5 Vita-Salute San Raffaele University, Milan, Italy

**Keywords:** Multiple sclerosis, COVID-19, SARS-CoV-2 vaccination, Fingolimod, Ocrelizumab

## Abstract

**Background:**

Recent observations suggest a lack of humoral response after SARS-CoV-2 vaccination in multiple sclerosis (MS) patients treated with fingolimod or ocrelizumab

**Objectives:**

To assess serological response to SARS-CoV-2 vaccination in MS patients receiving these disease-modifying treatments (DMTs) in a real-life setting.

**Methods:**

Retrospective clinical data collection from MS patients followed at San Raffaele Hospital MS Centre (Milan, Italy). All patients treated with fingolimod or ocrelizumab who had received a complete anti-COVID-19 vaccination course, with no clinical history suggestive of previous SARS-CoV-2 infection and with an available post-vaccination serological assay obtained at least 14 days after vaccination completion were considered for the study.

**Results:**

We collected data from 32 MS patients, 16 treated with fingolimod and 16 receiving ocrelizumab. Among the fingolimod group 10 patients (62.5%) had a positive serological response after vaccination and among ocrelizumab-treated patients a positive serological test was found in six cases (37.5%). No relation between serological response and clinical features (i.e., treatment duration, time between vaccination and last treatment dose, and white blood cells count) was identified.

**Conclusions:**

Our initial real-life experience suggests a variable antibody production in MS patients receiving these DMTs. At present, there are no sufficient data to do not recommend anti-SARS-CoV-2 vaccine in these patients.

SARS-CoV-2 infection has been responsible of an unprecedented pandemic all over the world, with consequences on several social aspects. Several concerns have been raised in relation to an increased susceptibility to COVID-19 in patients affected by Multiple sclerosis (MS), considering the many potential interactions with the immune system, disease-modifying treatments (DMTs) and neurological complications described in association with SARS-CoV-2 infection [1]. Recent reviews suggest MS itself does not seem to be associated with an increased severity of such an infection [2, 3], with a possible alert for anti-CD20 therapies [4]. Nevertheless, as in the general population, it remains fundamental to predispose adequate preventive strategies, including the recent availability of COVID-19 vaccines.

Inactivated vaccines are generally safe in MS, with the majority of data deriving from previous experiences with flu vaccines [5]. Despite specific information is not yet fully available from clinical trials, no specific safety concerns have emerged for both mRNA and viral vector SARS-CoV-2 vaccines in MS patients [6, 7]. COVID-19 vaccination is therefore highly recommended by national societies’ guidelines and institutions all over the world. However, it remains to be established whether COVID-19 vaccines are effective in MS, in particular in relation to possible interactions with ongoing DMTs. In this perspective first-line therapies (interferon-beta, glatiramer acetate, teriflunomide and dimethylfumarate) and natalizumab are not expected to compromise vaccine efficacy, while cell-depleting agents (ocrelizumab, rituximab, alemtuzumab and, at least in part, cladribine) as well as modulators of sphingosine–1–phosphate receptor, such as fingolimod, are believed to impact on immune responses after vaccination.

Post-marketing experiences in the field are gradually becoming available. Achiron and colleagues recently assessed humoral response to mRNA COVID-19 vaccines on an Israeli cohort of 93 MS patients-receiving second-line DMTs (23 cladribine, 26 fingolimod and 44 ocrelizumab) and 32 untreated MS patients [8]. After vaccination, a particularly low prevalence of positive SARS-CoV-2 IgG antibodies was detected in ocrelizumab (22.7%) and fingolimod (3.8%) groups. It was, therefore, proposed to avoid COVID-19 vaccination in fingolimod-treated patients (considering possible switch to other DMTs) and to schedule vaccination at least 9 months after last treatment dose in patients receiving ocrelizumab. This approach, although very conservative, opens to significant implications about disease activity, with a potential significant risk of relapse deriving from treatment discontinuation or delayed administration. A good serological response was instead detected among cladribine-treated patients (100%), thus confirming preliminary data available for flu vaccine [9].

We report our initial clinical experience, with the aim to investigate COVID-19 vaccines response in MS patients receiving ocrelizumab or fingolimod. We retrospectively collected data from 32 MS patients followed at San Raffaele Hospital MS Centre (Milan, Italy), 16 treated with fingolimod and 16 with ocrelizumab. All patients received a complete anti-SARS-CoV-2 vaccination course, consisting of two separate doses (30 with BNT162b2 mRNA vaccine produced by Pfizer BioNTech, two with mRNA-1273 vaccine produced by Moderna) and no one had a clinical history suggestive of previous SARS-CoV-2 infection. Anti-Spike protein antibody test was obtained at least 2 weeks (mean 33.1 days, range 14–71) after vaccination course completion. Serology tests have been performed in different laboratories as part of routine clinical assessment in order to evaluate humoral responses to vaccination (detailed techniques for each single patient are reported in Tables 1 and 2). Among the fingolimod group, 10 patients (62.5%) had a positive serological response after vaccination and among ocrelizumab-treated patients a positive serological test was found in 6 cases (37.5%). Complete clinical information is available in Tables 1 and 2; no relation between serological response and clinical features (i.e., treatment duration, time between vaccination and last treatment dose, and white blood cells count) was identified.

**Table 1:** Clinical information and serology of fingolimod-treated group

Patient	Sex	Age (years)	Disease duration (years)	EDSS	Vaccine	Test method	Antibody titer (U/ml)	Titer cut-off (U/ml)	Test result	Time vaccine-test (days)	WBC (× 10^9^/l)	Lymphocytes (× 10^9^/l)	Therapy duration	Previous IS
1	F	36.6	12.5	1.5	BNT162b2	CLIA	0.40	1.00	Negative	48	4.50	0.50	7.26	No
2	F	68.4	27.9	4.5	BNT162b2	ECLIA	64.29	0.80	Positive	36	2.90	0.40	3.16	No
3	F	57.8	19.0	3.5	BNT162b2	CLIA	4.40	7.10	Negative	23	5.31	0.55	8.01	No
4	M	26.9	6.0	1.0	BNT162b2	CLIA	58.00	NA	Positive	16	4.64	0.45	4.90	No
5	F	33.2	5.9	1.0	BNT162b2	CLIA	1.85	10.00	Negative	14	2.54	0.35	3.60	No
6	M	33.2	8.9	2.0	BNT162b2	CLIA	4.10	0.80	Positive	59	3.84	0.32	6.44	Yes
7	F	47.8	14.0	1.5	BNT162b2	ECLIA	0.74	NA	Negative	44	3.36	0.99	4.09	No
8	F	48.9	23.0	2.0	BNT162b2	CLIA	56.20	15.00	Positive	17	2.04	0.31	8.58	Yes
9	M	49.6	4.3	2.5	BNT162b2	CLIA	181.00	15.00	Positive	14	6.44	0.95	1.35	Yes
10	F	42.2	8.0	1.5	BNT162b2	CLIA	448.00	33.80	Positive	45	3.20	0.40	2.82	No
11	F	53.9	22.0	6.0	BNT162b2	CLIA	0.00	NA	Negative	37	6.59	0.87	5.96	No
12	M	47.4	23.9	1.5	BNT162b2	CLIA	107.30	50.00	Positive	40	3.80	0.38	1.91	No
13	F	40.9	17.3	1.5	BNT162b2	CLIA	7.13	1.00	Positive	16	5.93	0.55	6.56	No
14	F	18.2	5.1	1.0	mRNA-1273	CLIA	169.00	15.00	Positive	43	5.80	0.76	2.63	No
15	F	40.2	2.4	1.5	BNT162b2	CMIA	1.20	7.10	Negative	21	3.50	0.30	1.93	No
16	F	57.3	20.0	6.5	BNT162b2	CMIA	14.70	7.10	Positive	71	5.04	0.40	4.49	No

*WBC* white blood cells count, *IS* immunosuppressants—i.e. cyclophosphamide, mitoxantrone, *ECLIA* electrochemiluminescence immunoassay, *CLIA* chemiluminescence immunoassay, *CMIA* chemiluminescence microparticle immunoassay

**Table 2:** Clinical information and serology of ocrelizumab-treated group

Patient	Sex	Age (years)	Disease duration (years)	EDSS	Vaccine	Test method	Antibody titer (U/ml)	Titer cut-off (U/ml)	Test result	Time vaccine-test (days)	WBC (× 10^9^/l)	Lymphocytes (× 10^9^/l)	CD20 count (%)	Therapy duration (years)	Therapy cycles	Time vaccine-last dose (months)	Previous IS
1	F	50.6	13.4	5.5	BNT162b2	ECLIA	< 0.40	0.80	Negative	37	5.76	0.99	0.00	1.3	2	4.8	Yes
2	F	30.8	6.8	1.0	BNT162b2	CMIA	2.80	50.00	Negative	33	NA	NA	NA	2.1	4	2.4	No
3	F	40.0	14.0	3.5	BNT162b2	CMIA	0.30	NA	Negative	25	4.64	1.22	1.00	2.5	4	5.0	No
4	F	41.1	22.4	5.5	BNT162b2	DELFIA	21.00	1.20	Positive	61	NA	NA	NA	2.0	2	8.2	Yes
5	F	50.8	8.3	6.5	BNT162b2	ECLIA	98.50	0.80	Positive	19	6.37	1.60	11.10	3.4	5	5.4	Yes
6	M	57.1	8.4	5.0	BNT162b2	ECLIA	238.00	0.80	Positive	31	6.30	1.30	2.10	3.4	6	5.0	Yes
7	F	28.0	3.4	1.5	BNT162b2	CLIA	0.05	1.00	Negative	31	6.60	1.90	0.03	2.4	4	3.1	No
8	F	61.3	11.0	6.0	BNT162b2	ECLIA	0.11	1.10	Negative	34	5.28	2.35	0.10	3.2	6	5.8	No
9	F	46.6	26.0	6.5	BNT162b2	CLIA	0.07	10.00	Negative	66	NA	NA	NA	2.7	6	2.8	Yes
10	M	32.3	14.0	6.5	BNT162b2	ECLIA	< 0.40	0.80	Negative	30	9.20	2.20	0.00	2.5	5	5.0	No
11	M	23.0	6.1	2.5	mRNA-1273	ECLIA	< 0.40	0.80	Negative	42	6.50	2.30	0.50	2.9	5	4.6	No
12	F	56.3	1.3	4.5	BNT162b2	CLIA	65.90	15.00	Positive	19	4.20	1.60	0.50	0.0	1	3.5	No
13	M	43.4	4.3	4.5	BNT162b2	ECLIA	< 0.40	0.80	Negative	18	5.80	1.20	0.00	2.7	5	5.9	No
14	M	36.8	10.9	3.5	BNT162b2	ECLIA	22.40	0.80	Positive	21	5.49	1.18	0.03	2.1	4	5.9	No
15	F	64.1	19.5	3.0	BNT162b2	CMIA	155.00	33.80	Positive	21	3.96	1.12	0.04	2.1	4	5.5	Yes
16	M	43.2	25.2	4.0	BNT162b2	CMIA	< 2.20	22.00	Negative	28	4.33	1.14	0.03	2.2	4	4.2	Yes

*WBC* white blood cells count, *IS* immunosuppressants—i.e. cyclophosphamide, mitoxantrone, *ECLIA* electrochemiluminescence immunoassay, *CLIA* chemiluminescence immunoassay, *CMIA* chemiluminescence microparticle immunoassay, *DELFIA* dissociation-enhanced lanthanide fluorescent immunoassay

Our experience, in comparison to previously published data [8], suggests that humoral response to SARS-CoV-2 vaccination might be highly variable, even in patients treated with fingolimod or ocrelizumab. As a consequence, we believe that SARS-CoV-2 vaccination should be recommended also in MS patients treated with such agents. Indeed, available data are still too limited to suggest treatment discontinuation in order to favor a vaccination response, considering the significant risk of clinical relapse and MRI activity associated to second-line treatment withdrawal (at least in relapsing–remitting MS patients). Furthermore, initial reports relative to other medical conditions causing immunodeficiency suggest the possibility of an efficient cell-mediated immunity after vaccination even in the absence of a detectable humoral response [10].

Clearly, this study is not without limitations: the sample size is relatively small and data collection is retrospective, with serological exams performed with different techniques. In addition, we do not have pre-vaccinations serological tests available; northern Italy had a very high prevalence of SARS-CoV-2 infection over the last year, therefore we cannot exclude a previous asymptomatic infection possibly influencing the serological response.

In this historical moment, it is of outmost importance that a very large proportion of the population, including people with MS, adheres to mass vaccination campaigns, in order to get through the current pandemic condition. With the rapid progression of SARS-CoV-2 vaccination programs all over the world, more extensive real-life data from different geographic regions are likely to become available in the near future. The assessment, in the context of prospective clinical trials, of humoral and in particular T cell response to SARS-CoV-2 vaccination will be also crucial to tailor the clinical management of MS patients.
